# High- and Low-Affinity Transport in Plants From a Thermodynamic Point of View

**DOI:** 10.3389/fpls.2019.01797

**Published:** 2020-01-30

**Authors:** Ingo Dreyer, Erwan Michard

**Affiliations:** ^1^Centro de Bioinformática y Simulación Molecular, Facultad de Ingeniería, Universidad de Talca, Talca, Chile; ^2^Cell Biology and Molecular Genetics, University of Maryland, College Park, College Park, MD, United States

**Keywords:** computational cell biology, dual-affinity transport, high-affinity transport, low-affinity transport, modelling, nutrient transport, plant biophysics

## Abstract

Plants have to absorb essential nutrients from the soil and do this *via* specialized membrane proteins. Groundbreaking studies about half a century ago led to the identification of different nutrient uptake systems in plant roots. Historically, they have been characterized as “high-affinity” uptake systems acting at low nutrient concentrations or as “low-affinity” uptake systems acting at higher concentrations. Later this “high- and low-affinity” concept was extended by “dual-affinity” transporters. Here, in this study it is now demonstrated that the affinity concept based on enzyme kinetics does not have proper scientific grounds. Different computational cell biology scenarios show that affinity analyses, as they are often performed in wet-lab experiments, are not suited for reliably characterizing transporter proteins. The new insights provided here clearly indicate that the classification of transporters on the basis of enzyme kinetics is largely misleading, thermodynamically in no way justified and obsolete.

## Introduction

For more than about half a century the scientific description of nutrient transport in plants has been dominated by the terminology of “high- and low-affinity” transport processes. Historically it goes back to the pioneering work of Emanuel Epstein and co-workers (Epstein et al., 1963). In their groundbreaking study, Epstein and colleagues could resolve two distinct mechanisms of potassium absorption by barley roots. In the absence of knowledge on channels and transporters, the nutrient fluxes were described in analogy to classical enzyme kinetics (Epstein and Hagen, 1952). This theoretical concept employing the Michaelis-Menten equation described the measurement data very satisfactorily and allowed to separate a low concentration uptake mechanism 1 (called “high-affinity”) from a high concentration uptake mechanism 2 (called “low-affinity”). From then on, this concept began its triumphal march and was adopted by the scientific community largely without reflection. In the course of the molecular revolution with the cloning and molecular characterization of channels and transporters, it even developed a life of its own. Besides the categorization into either “high-affinity” or “low-affinity” uptake systems (Rodríguez-Navarro and Rubio, 2006; Sharma et al., 2013), some cloned transporters were assigned “dual affinities” (Fu and Luan, 1998; Kim et al., 1998; Liu et al., 1999). It was also suggested that post-translational modifications could switch between the affinity modes (Liu and Tsay, 2003). The “high-affinity/low-affinity” jargon suggests that the transported nutrient binds better or less well to the respective transporter protein. As a consequence, “high-affinity” transporters are assumed to act better at low concentrations while at higher nutrient concentrations they are saturated in contrast to “low-affinity” transporters.

In this article it will be shown that all these far-reaching interpretations made on the basis of the historical enzyme kinetics concept are incorrect. Here, computationally assisted thought experiments are carried out exemplarily for K^+^ channels and proton-coupled K^+^ transporters with well-defined properties. H^+^-coupled K^+^ co-transporters are widely accepted as “high-affinity” transporters (Quintero and Blatt, 1997; Santa-María et al., 1997; Fu and Luan, 1998; Kim et al., 1998); K^+^ channels are widely accepted as “low-affinity” transporters (Anderson et al., 1992; Schachtman et al., 1992; Sentenac et al., 1992). Different scenarios show that affinity analyses, as they are often performed in wet-lab experiments, are not suited for reliably characterizing transporter proteins.

## Method

### Mathematical Description of Transporter Activities

Irrespective of the exact transport mechanism, both K^+^ channels and proton-coupled K^+^ transporters can be considered as diffusion facilitators. A K^+^ channel mediates the selective transport of K^+^ along the transmembrane electrochemical gradient for potassium, while a 1:1 K^+^/H^+^ co-transporter allows the flux along the combined K^+^/H^+^ gradient (Rodriguez-Navarro et al., 1986). In its general form, the current through *N* open K^+^ channels or *N* active K^+^/H^+^ transporters can be described by

(1) $$I_{X}(V)=N\cdot z_{X}\cdot e_{0}\cdot j_{X}(V)\cdot A$$

where *e_0_* ≈ 1.602×10^–19^*C* is the elementary charge, *A* the surface of a cross-section of the channel/transporter, *z_X_* the valence of the transport, and *j_X_*(*V*) the flux density of ions per second per surface unit; *X* represents the respective transporter type (K, *z_K_* = 1, or K/H, *z_K/H_* = 2). The current and the flux density are zero at *V_rev_X_*, the voltage at which the electrochemical driving force is zero: *I_X_*(*V_rev_X_*) = *j_X_*(*V_rev_X_*) = 0. This fact can be used to simplify equation (1) by its first-order Taylor approximation:

(2) $$I_{X}(V)\approx I_{X}(V_{rev\_X})+(V-V_{rev\_X})\cdot {\frac{dI_{X}(V)}{dV}|}_{V=V_{rev\_X}}=g_{X}\cdot (V-V_{rev\_X})$$

with the membrane conductance of the respective transporter type:

(3) $$g_{X}=N\cdot z_{X}\cdot e_{0}\cdot A\cdot {\frac{dj_{X}(V)}{dV}|}_{V=V_{rev\_X}}$$

Higher order terms do not contribute significantly [*σ*^2^≈0], as *e.g.* the linear current-voltage properties of single open ion channels show. Equation (2) describes the current through open channels/transporters. Usually, these transporters are regulated by the environmental conditions, e.g. by the membrane voltage. This dependency can be included by a factor *p_X_* that represents the probability of the channel/transporter being active/open resulting in the conductance *G_X_*:

(4) $$G_{X}=g_{X}\cdot p_{X}$$

At *V* = *V_rev_X_* the driving energy gradient Δ*µ_X_* is zero. Δ*µ_X_* is the difference between the electrochemical potentials inside and outside the cell:

(5) $$\Delta \mu_{K}=RT\cdot \ln\left(\frac{{\left[{\text{K}}^{+}\right]}_{int}}{{\left[{\text{K}}^{+}\right]}_{ext}}\right)+F\cdot \left(\psi_{int}-\psi_{ext}\right)$$

(6) $$\Delta \mu_{K/H}=RT\cdot \ln\left(\frac{{\left[{\text{K}}^{+}\right]}_{int}\cdot {\left[{\text{H}}^{+}\right]}_{int}}{{\left[{\text{K}}^{+}\right]}_{ext}\cdot {\left[{\text{H}}^{+}\right]}_{ext}}\right)+2\cdot F\cdot \left(\psi_{int}-\psi_{ext}\right)$$

Here, [K^+^]*_int_*, [K^+^]*_ext_*, [H^+^]*_int_*, and [H^+^]*_ext_* are the potassium and proton concentrations inside and outside the cell, respectively, *ψ* the electric potential (*V* = *ψ_int_* - *ψ_ext_*), *F* the Faraday constant, *R* the gas constant, and *T* the absolute temperature. At equilibrium (Δ*µ_X_* = 0), the equilibrium voltages (*V_rev_*) of the two transporter types are (Nernst-Equations):

(7) $$V_{rev\_K}=E_{K}=\frac{RT}{F}\cdot \ln\left(\frac{{\left[{\text{K}}^{+}\right]}_{ext}}{{\left[{\text{K}}^{+}\right]}_{int}}\right)$$

(8) $$V_{rev\_K/H}=E_{K/H}=\frac{RT}{2F}\cdot \ln\left(\frac{{\left[{\text{K}}^{+}\right]}_{ext}\cdot {\left[{\text{H}}^{+}\right]}_{ext}}{{\left[{\text{K}}^{+}\right]}_{int}\cdot {\left[{\text{H}}^{+}\right]}_{int}}\right)$$

Consequently, the transmembrane current *via* potassium channels is represented by:

(9) $$I_{K}\left(V\right)=G_{K}\cdot \left(V-\frac{RT}{F}\cdot \ln\left(\frac{{\left[K^{+}\right]}_{ext}}{{\left[K^{+}\right]}_{\mathrm{int}}}\right)\right)$$

And the current *via* 1:1 proton-coupled potassium transporters is mathematically described by:

(10) $$I_{H/K}\left(V\right)=G_{K/H}\cdot \left(V-\frac{RT}{2F}\cdot \ln\left(\frac{{\left[K^{+}\right]}_{ext}\cdot {\left[H^{+}\right]}_{ext}}{{\left[K^{+}\right]}_{\mathrm{int}}\cdot {\left[H^{+}\right]}_{\mathrm{int}}}\right)\right)$$

The conductances *G_K_* and *G_K/H_* are in fact composite parameters [see equations (3–4)], which gather all dependencies on a variety of environmental parameters. Their values may change with the substrate concentration (via *p_X_*) and may show saturation, or they may be regulated by the membrane voltage (via *p_X_*), by gene expression (via *N*), and/or downstream signaling cascades (via *N*, *p_X_*). Thus, the equations (9) and (10) cover all biological scenarios for these transporter types in a generalized way.

The action of the H^+^-ATPase was mechanistically described by a six-state model (Dreyer, 2017), which is mathematically represented by a sigmoidal function (Gajdanowicz et al., 2011). Under normal physiological conditions (pH_int_ 7.0) an appropriate mathematical description for the dependency on the voltage and [H^+^]_ext_ is:

(11) $$I_{pump}\left(V\right)=I_{\max}\cdot \frac{1}{1+\frac{{\left[H^{+}\right]}_{ext}}{100\mu M}}\cdot \frac{1\;-\;\frac{{\left[H^{+}\right]}_{ext}}{0.1\mu M}\;\cdot \;e^{-\left(\frac{F}{RT}\cdot V+12.2\right)}}{1+e^{-\left(\frac{F}{RT}\cdot V+8\right)}+e^{-\left(0.32\cdot \frac{F}{RT}\cdot V+2.5\right)}}$$

Here, *I_max_* denotes the maximal current at very positive voltages at low [H^+^]_ext_. The pump current amplitude decreases with increasing [H^+^]_ext_ and the voltage

(12) $$V_{rev\_pump}=\frac{RT}{F}\cdot \left[\ln\left(\frac{{\left[H^{+}\right]}_{ext}}{0.1\mu M}\right)-12.2\right]$$

at which the energy from ATP-hydrolysis reaches its limit for pumping [*I_pump_*(*V_rev_*) = 0], shifts to less negative values. The numeric values in the equations (11) and (12) are empirical constants to describe the experimentally observed dependencies.

### Computational Cell Biology

Following the mathematical description of all transporters, an *in silico* cellular system was programmed and computational cell biology (dry-laboratory) experiments were performed using the VCell Modeling and Analysis platform developed by the National Resource for Cell Analysis and Modeling, University of Connecticut Health Center (Loew and Schaff, 2001).

### Michaelis-Menten Kinetics

To the present day, transport processes in plants are often described with the Michaelis-Menten kinetics developed for enzyme-catalyzed reactions. The reaction scheme

$$E+S\;\;\;\begin{array}{c} k_{1}\\ ⇄\\ k_{2} \end{array}\;\;\;ES\;\;\;\begin{array}{c} k_{3}\\ ⇄\\ k_{4} \end{array}\;\;\;E+P$$

is interpreted in the way that *E* is the free transporter, *ES* is the transporter with the bound nutrient, *S* is the transported nutrient at one side of the membrane (e.g. *S* = [K^+^]_ext_), and *P* the nutrient at the other side (e.g. *P* = [K^+^]_int_). In equilibrium $\left(\frac{dES}{dt}=\frac{dE}{dt}=0\right)$ the flux from the external to the internal medium is given by:

(13) $$J=N\cdot \frac{k_{1}\cdot k_{3}\cdot {\left[K^{+}\right]}_{ext}-k_{2}\cdot k_{4}\cdot {\left[K^{+}\right]}_{\mathrm{int}}}{k_{2}+k_{3}+k_{1}\cdot {\left[K^{+}\right]}_{ext}+k_{4}\cdot {\left[K^{+}\right]}_{\mathrm{int}}}$$

Equation (13) is applied in plant biology almost exclusively with the approximation *k*_4_≈0-irrespective of the fact that this approximation is incompatible with the Nernst-Equation – yielding a Michaelis-Menten-type equation:

(14) $$J\approx J_{\max}\cdot \frac{{\left[K^{+}\right]}_{ext}}{K_{m}+{\left[K^{+}\right]}_{ext}}$$

with *J_max_* = *N* × *k_3_* and *K_m_* = (*k_2_* + *k_3_*)/*k_1_*. In affinity analyses, equation (14) is employed alone or as a sum of two components, a “high-affinity” and a “low-affinity” component:

(15) $$J=J_{\max,high}\cdot \frac{{\left[K^{+}\right]}_{ext}}{K_{m,high}+{\left[K^{+}\right]}_{ext}}+J_{\max,low}\cdot \frac{{\left[K^{+}\right]}_{ext}}{K_{m,low}+{\left[K^{+}\right]}_{ext}}$$

## Results

### Apparent Affinities Can Be an Artifact of Data Display

**Figure 1:**
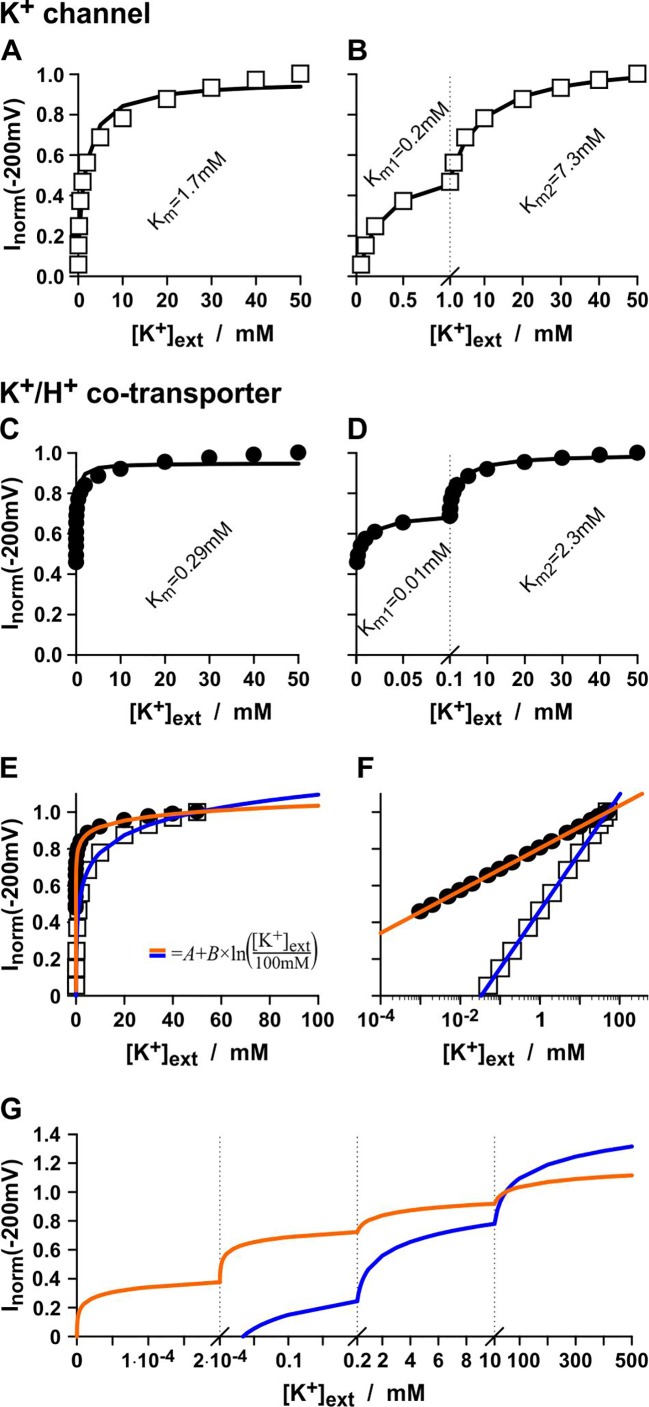
The pitfalls of affinity analyses. Same data, different display = different affinities? Simulated experiments with a K^+^-independent K^+^ channel **(A, B)** and a K^+^-independent K^+^/H^+^ 1:1 co-transporter **(C, D)**. Channel/transporter-mediated currents were determined at *V* = -200 mV with [K^+^]_int_ = 100 mM, pH_int_ = 7.0, pH_ext_ = 5.0 at varying [K^+^]_ext_. Currents were normalized to the values obtained with [K^+^]_ext_ = 50 mM. The same data were displayed linearly in the interval from 0 to 50 mM **(A, C)**, linearly split into the intervals from 0 to 1 mM and from 1 to 50 mM **(B)**, and linearly split into the intervals from 0 to 0.1 mM and from 0.1 to 50 mM **(D)**. The solid lines represent best fits with a Michaelis-Menten-equation [equation (14)] in the respective interval with the indicated K_m_-values. **(E, F)** Fit of the data from A and C with logarithmic functions of the type *I = A*+*B*×ln([K^+^]_ext_/100mM). For the K^+^ channel (*white squares*) the fit parameters were *A* = 1.095 and *B* = 0.137 (*blue line*), while for the K^+^/H^+^ 1:1 co-transporter (black dots) the parameters were *A* = 1.035 and *B* = 0.05 (*orange line*). **(G)** Split-display of the fit curves from E and F in linear intervals (i) from 0 to 0.2 µM, (ii) from 0.2 µM to 0.2 mM, (iii) from 0.2 mM to 10 mM, and (iv) from 10 mM to 500 mM.

Affinity analyses can be full of pitfalls. To illustrate this, a common voltage-clamp experiment as it can be found often in literature to characterize plant transporters has been computationally simulated: The currents flowing through a K^+^ channel or a K^+^/H^+^ co-transporter were measured at a fixed membrane voltage with a constant internal K^+^ concentration ([K^+^]_int_ = 100mM) under varying external K^+^. In the simulations, K^+^ channels and K^+^/H^+^ transporters were chosen that do *not* change their conductance with [K^+^]_ext_, *i.e. G_K_* and *G_K/H_* are K^+^-*in*dependent and have both the same constant value. Nevertheless, the “current vs. [K^+^]_ext_ display” suggests an apparent K^+^-affinity of the K^+^ channel of K_m_ = 1.7mM (**Figure 1A**) and of the K^+^/H^+^ co-transporter of K_m_ = 0.29mM (**Figure 1C**) when analyzed with the Michaelis-Menten formalism. Surprisingly, when displaying the same data in a different manner, both could be assigned even an apparent dual affinity. The K^+^ channel was now characterized by a high-affinity component of K_m_ = 0.2mM and a low-affinity component of K_m_ = 7.3mM (**Figure 1B**), while the K^+^/H^+^ co-transporter had a high-affinity component of K_m_ = 0.01mM and a low-affinity component of K_m_ = 2.3mM (**Figure 1D**).

It should be emphasized again that in the simulation both, K^+^ channel and K^+^/H^+^ co-transporter, are *in*dependent of [*K^+^*]*_ext_*. In fact, the apparent affinities originate from the [*K^+^*]*_ext_*-dependent change in *V_rev_* [equations (7-10)]. If the data were displayed on a log-[K^+^]_ext_ scale they could be described by straight lines representing linear functions of the type *I*=*A*+*B*×ln([*K*^+^]*_ext_*) (**Figures 1E, F**). Were these curves presented in different linear intervals, as done in affinity analyses, several seemingly saturating affinity curves could be separated (**Figure 1G**). Thus, the K_m_ values obtained from the affinity analyses are artifacts of the data display and have no significant meaning.

Consequently, a similar K_m_-analysis of real channels or transporters (with real experimental data) is meaningless and might be largely misguiding. Still, the simulations show that there are differences between the K^+^ channel and the K^+^/H^+^ co-transporter. The apparent K_m_-values, albeit misleading and without special meaning, are always smaller for the co-transporter than for the channel, despite the fact that *G_K_ = G_K/H_*. The K_m_-analysis is therefore a wrongly used tool that yet allows a distinction between the transporter types. Nevertheless, the widely used classification into “high-affinity” (= low K_m_ values) and “low-affinity” (= high K_m_ values) transporters becomes untenable (Dreyer, 2017) due to these new insights. They further raise the question, what is then the real difference between the K^+^ transporter types from a biophysical and physiological point of view, if it is not the affinity of the proteins towards K^+^?

### The Features of the Proton Pump Lead to Believe in High- and Low-Affinity K^+^ Transport

**Figure 2:**
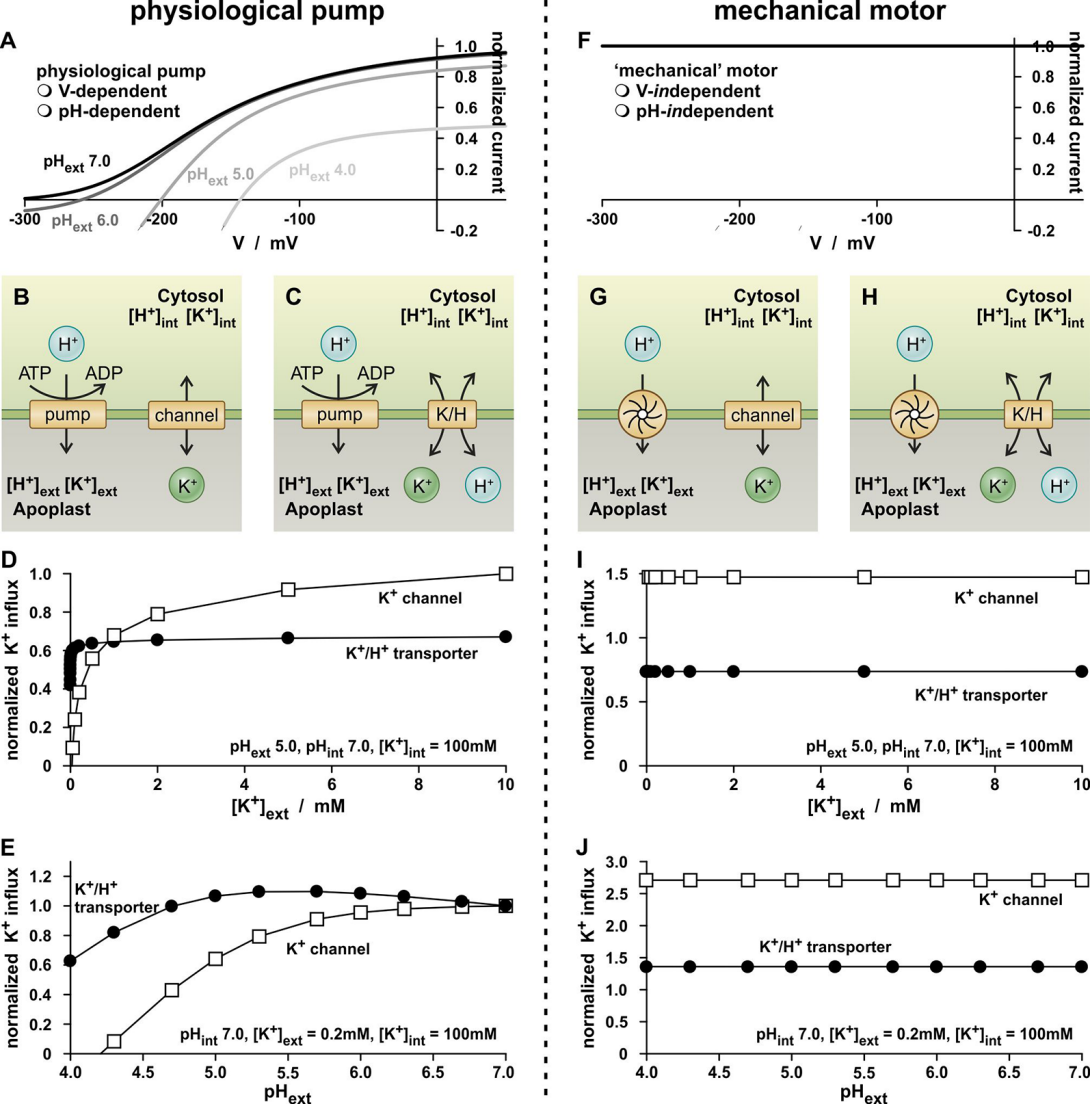
Simple physiological K^+^-uptake circuits. **(A–E)** K^+^-uptake circuits fueled by a physiological proton ATPase. **(A)** As a consequence of the pump cycle driven by the energy from ATP hydrolysis, the activity of the proton pump inevitably depends on the membrane voltage (V) and the proton concentrations on both sides of the membrane. Shown is the current-voltage characteristic of the pump current [equation (11)] for pH_int_ 7.0 and four different pH_ext_ values. **(B, C)** Schematic representation of two simple K^+^-uptake modules. A (pH- and voltage-dependent) proton pump energizes the uptake of potassium *via* a K^+^ channel **(B)** and *via* a K^+^/H^+^ 1:1 co-transporter **(C)**. **(D, E)** Dependency of the K^+^ flux mediated by the two modules on the external K^+^ concentration **(D)** and on the external pH **(E)**. Please note that for both modules, the environmental conditions were the same and *G_K_* = *G_K/H_*. The observed differences thus result from the different transport mechanisms of the channel/transporter. Flux values were normalized to the K^+^ flux mediated by the channel based module at [K^+^]_ext_ = 10 mM **(D)**, and pH_ext_ 7.0 **(E)**. **(F–J)** K^+^-uptake circuits fueled by a voltage- and pH-*in*dependent proton motor. **(F)** The mechanical motor is independent of the voltage and the pH. **(G, H)** Schematic representation of two simple K^+^-uptake modules. A mechanical proton motor energizes the uptake of potassium *via* a K^+^ channel **(G)** and *via* a K^+^/H^+^ 1:1 co-transporter **(H)**. **(I, J)** Dependency of the K^+^ flux mediated by the two modules on the external K^+^ concentration **(I)** and on the external pH **(J)**. Please note that for both modules, the environmental conditions were the same and *G_K_* = *G_K/H_*. The observed differences thus result from the different transport mechanisms of the channel/transporter.

To assess the former question, another thought experiment has been carried out with the K^+^-independent K^+^ channel and K^+^/H^+^ co-transporter. This time the transporters were combined with a H^+^-ATPase (**Figure 2A**), which energizes the K^+^-uptake (**Figures 2B, C**), in order to approach further the physiological conditions and to simulate common K^+^ uptake experiments. The concentrations ([*H^+^*]*_int_*, [*K^+^*]*_int_*, [*H^+^*]*_ext_*, [*K^+^*]*_ext_*) were kept constant and the membrane voltage was allowed to relax freely until a stabile equilibrium has been established. This occurred almost instantaneously within a few milliseconds. In this condition, the pumped protons compensate electrically the fluxes through the K^+^-transporters. The magnitude of the steady-state fluxes depends on the ionic conditions on both sides of the membrane.

In a first experiment, the dependency of the steady state K^+^ fluxes on the external potassium concentration was screened (**Figure 2D**). For both transporter modules, hyperbolic curves were obtained that saturated for the K^+^/H^+^ transporter at smaller [*K^+^*]*_ext_* than for the K^+^ channel. This result fueled again the interpretation that the K^+^/H^+^ transporter was a “high-affinity” transporter while the K^+^ channel was a “low-affinity” transporter, despite the fact that both were K^+^-*in*dependent in the thought experiment. However, also these data could be displayed as in **Figures 1B, D** in two different concentration intervals and would characterize both as “dual-affinity” K^+^ transporters (not shown).

The concept of “high- and low-affinity K^+^ transport” got more cracks in the analysis of the dependency of the K^+^ fluxes on the external proton concentration. In contrast to the proton-coupled K^+^ transport *via* the K^+^/H^+^ co-transporter, the flux through the K^+^ channel should actually be independent of [*H^+^*]*_ext_*. However, this was not the case. Instead, the channel-mediated K^+^ flux declined with increasing [*H^+^*]*_ext_* (**Figure 2E**). The K^+^ flux through the K^+^/H^+^ 1:1 co-transporter showed a bi-phasic behavior: as pH_ext_ dropped from 7.0 to 5.5, the K^+^ flux increased slightly, while it decreased as the pH level dropped further. Thus, the K^+^ fluxes are not determined by the activity of the K^+^ transporters, alone.

Indeed, an in-depth analysis revealed that the apparent affinities are in fact not a feature of the K^+^-transporters but were introduced into the system by the voltage- and pH-dependency of the H^+^-ATPase. This became evident when replacing the physiological proton pump by a mechanical motor that is independent of the membrane voltage and the proton concentrations on both sides of the membrane (**Figures 2F–J**). The mechanical motor exported always the same number of protons per time irrespective of the voltage, pH_ext_ and pH_int_ (**Figure 2F**). The replacement of the pump eliminated the hyperbolic K^+^-uptake kinetics for both transporter types (**Figures 2I, J**). The K^+^ fluxes were independent of changes in [*K^+^*]*_ext_* and [*H^+^*]*_ext_*, as it should be expected for transporters with [K^+^]- and [H^+^]-independent conductances, *G_K_* and *G_K/H_*.

Thus, K^+^ flux measurements in our thought experiments as well as those in real wet-lab experiments are strongly influenced by the properties of the energizing proton-pump. The assigned affinities are therefore strongly misguiding and without any significant meaning for the K^+^ transporter proteins.

### The Real Difference Between K^+^ Channels and K^+^/H^+^ Co-Transporters

**Figure 3:**
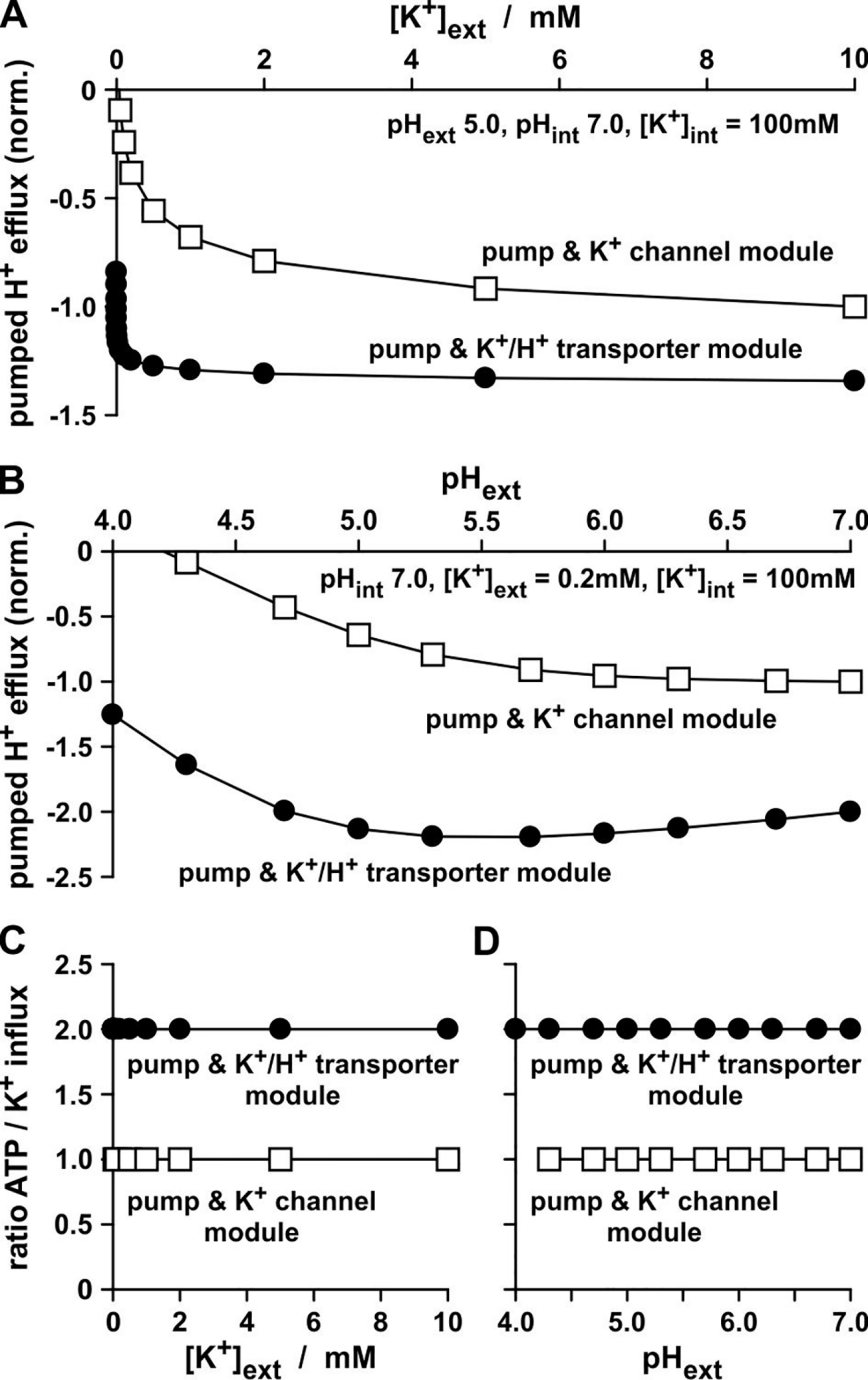
Proton pump activity during K^+^-uptake. **(A, B)** H^+^ flux *via* the pump measured in the *in-silico* K^+^-uptake experiments presented in Fig. 2D and E. The H^+^ fluxes were normalized to the K^+^ flux values measured for the channel-based module at [K^+^]_ext_ = 10 mM **(A)**, and pH_ext_ 7.0 **(B)**. The negative values indicate H^+^ effluxes in contrast to the K^+^ influxes presented in Fig. 2D and E. **(C, D)** For each pumped proton an ATP molecule has be to hydrolyzed. Ratios of the consumed ATP molecules per accumulated K^+^ ion in the *in-silico* experiments presented in **Figures 2D, E** and **3** **(A**, **B)**. To calculate the ratios, the absolute values of the data displayed in **(A)** were divided by the corresponding data from **Figure 2D** and the absolute values of the data displayed in **(B)** were divided by the corresponding data from **Figure 2E**.

The experiments presented in **Figure 2** were carried out with identical transporter conductances: *G_K_* = *G_K/H_*. That is why it is all the more amazing that the K^+^/H^+^ co-transporter transports just half the number of K^+^ ions per time-interval than the K^+^ channel (**Figures 2I, J**), despite the same energizing power source. Additionally, both modules, “pump & K^+^ channel” (**Figures 2B, G**) and “pump & K^+^/H^+^ co-transporter” (**Figures 2C, H**), do not differ in their net transfer ratios: both exchange 1 H^+^ for 1 K^+^. However, and this is the real crucial difference between the two modules, the K^+^/H^+^ co-transporter-based module pumped twice the number of protons per accumulated K^+^ (Rodriguez-Navarro et al., 1986) than the K^+^ channel-based module (**Figure 3**). Thus, the uptake of a K^+^ ion *via* the “pump & K^+^/H^+^ co-transporter” module consumed the hydrolysis of 2 ATP molecules, while the uptake *via* the “pump & K^+^ channel” module costed only 1 ATP molecule (**Figures 3C, D**).

**Figure 4:**
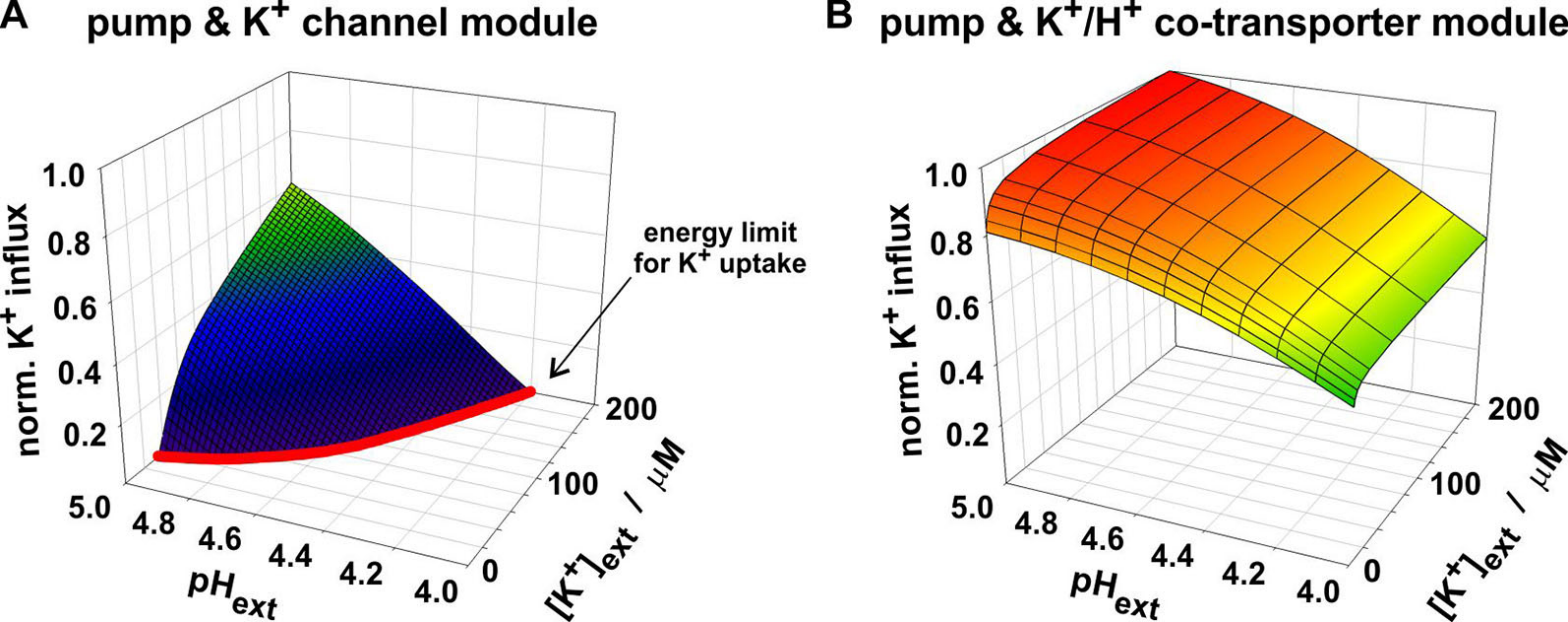
Dependency of the two compared K^+^ uptake modules on [K^+^]_ext_ and [H^+^]_ext_. Both modules mediate the net-antiport of K^+^ and H^+^ across the membrane. The module of proton pump and K^+^ channel **(A)** reaches its energy limit (*red line*) at conditions in which the co-transporter-based module still allows K^+^ uptake **(B)**. At very low [K^+^]_ext_ and/or high [H^+^]_ext_ the energy from ATP-hydrolysis is not sufficient anymore to energize the combined K^+^-uptake/H^+^-release *via* the pump & K^+^ channel module.

The advantage of the channel-based module was the “cheap” accumulation of K^+^; its disadvantage was its operating limit at low [*K^+^*]*_ext_* and high [*H^+^*]*_ext_*. To enable K^+^ uptake by a K^+^ channel, the membrane voltage needed to be more negative than *E_K_*, the equilibrium voltage for potassium [equation (7)], which depends on [*K^+^*]*_ext_*. For a 10-fold decrease in [K^+^]_ext_, E_K_ dropped by about 59 mV. Such a negative voltage could be established by the H^+^-ATPase if the energy from ATP-hydrolysis was sufficient to pump protons against their electrochemical gradient out of the cell. The higher [*H^+^*]*_ext_* was, however, the more difficulties the pump had to establish a sufficiently negative *V* (**Figure 2A**). As a consequence of both effects, the channel-based module reached the energy limit for K^+^ uptake already at moderate conditions (**Figure 4A**). In contrast, the co-transporter-based module still operated as a K^+^ uptake system under these conditions (**Figure 4B**). The reason was the coupling of the K^+^- and the H^+^-gradient. The equilibrium voltage of the K^+^/H^+^ 1:1 co-transporter [*E_K/H_*, equation (8)] dropped only by about 29.5 mV for a 10-fold decrease in [*K^+^*]*_ext_*. Additionally, *E_K/H_* raised with increasing [*H^+^*]*_ext_* which partially compensated the detrimental effect of [*H^+^*]*_ext_* on the pump. Thus, the real difference between K^+^ channels and K^+^/H^+^ co-transporters is not their affinity towards K^+^ rather than the energization of the transport process.

## Discussion

Solidly based thought experiments have an inestimable value in gaining new insights. They allow, for instance, to test conditions that are hard to achieve in conventional wet-laboratory experiments. In the present study, different K^+^ uptake systems were tested which were absolutely certain not to be dependent on [K^+^]. With these transporters the half-century old concept of “high- and low-affinity uptake systems” has been challenged in computer-aided dry laboratory experiments. Interestingly, the results of the experiment-mimicking simulations mirrored those obtained in wet-lab experiments with hyperbolic curves that saturated for the K^+^/H^+^ co-transporter at smaller [*K^+^*]*_ext_* than for the K^+^ channel. The usual interpretation of such a result is that the co-transporter is a “high-affinity uptake system” while the K^+^ channel is a “low-affinity uptake system”. The fact that in the simulations channel and co-transporter had both the same affinity towards [*K^+^*]*_ext_* clearly shows the absurdity of such an interpretation. Actually, the assigned affinities are not characteristics of the investigated transporters, but depend on several external parameters instead.

The equations (9) and (10) cover the mathematical description of the entire biological range of K^+^ channels and K^+^/H^+^ 1:1 co-transporters. The parameters *G_K_* and *G_K/H_*, respectively, provide individual channels/transporters with their unique particular flair. The experimental characterization of channels and transporters in the wet-lab usually aims at getting knowledge about these parameters and their dependency on environmental conditions. Here, we carried out simulations with idealized transporters with conductances *G_K_* and *G_K/H_* that did not depend on any environmental parameter. But even in this simplest case, the features of *G_K_* and *G_K/H_* could only be resolved in artificial conditions (**Figures 2F–J**). In all other dry-lab experiments mimicking conventional wet-lab experiments artifactual affinities were assigned to the transporters. In real experiments the conductance of a transporter might depend on a variety of environmental parameters. The value may change with the substrate concentration and may show saturation, or it may be regulated by the membrane voltage, by gene expression and/or downstream signaling cascades. The presented simulations indicate that all these dependencies cannot be reliably resolved in affinity analyses.

The original “high- and low-affinity concept” proves to be largely misleading. Its further evolution to “dual-affinity”, however, is an even more serious aberration. The examples presented in **Figure 1** illustrate clearly that an apparent “dual-affinity” can be “generated” just by a different display of the same data. Worse still, by further fragmentation of the x-axis it is possible to fake even a “triple or quadruple-affinity” (**Figure 1G**). Thus, the affinity analyses, as they are often performed in wet-lab experiments, are not suited for reliably characterizing transporter proteins. The classification of transporters on the basis of enzyme kinetics is largely misleading and obsolete.

## Data Availability Statement

All datasets generated for this study are included in the article/supplementary material.

## Author Contributions

ID conceived the project and prepared the original draft. EM and ID had intellectual input on the project and prepared the final manuscript.

## Funding

This work was supported partially by the Fondo para Proyectos de Investigación Enlace Fondecyt of the Universidad Talca to ID.

## Conflict of Interest

The authors declare that the research was conducted in the absence of any commercial or financial relationships that could be construed as a potential conflict of interest.
